# The Attitude towards Vaccination of Health Sciences Students at a Spanish University Improved over the First 18 Months of the COVID-19 Pandemic

**DOI:** 10.3390/vaccines10020237

**Published:** 2022-02-03

**Authors:** Francisco Javier Pérez-Rivas, Ramón Del Gallego-Lastra, Cristina Maria Alves Marques-Vieira, Candelas López-López, Silvia Domínguez-Fernández, Milagros Rico-Blázquez, María Julia Ajejas Bazán

**Affiliations:** 1 Departamento de Enfermería, Facultad de Enfermería, Fisioterapia y Podología, Universidad Complutense de Madrid, Plaza Ramón y Cajal no. 3, Ciudad Universitaria, 28040 Madrid, Spain; rgallego@ucm.es (R.D.G.-L.); canlopez@ucm.es (C.L.-L.); milarico@ucm.es (M.R.-B.); majejas@ucm.es (M.J.A.B.); 2 Grupo de Investigación UCM “Salud Pública-Estilos de Vida, Metodología Enfermera y Cuidados en el Entorno Comunitario”, Departamento de Enfermería, Facultad de Enfermería, Fisioterapia y Podología, Universidad Complutense de Madrid, 28040 Madrid, Spain; sildom01@ucm.es; 3 Grupo de Investigación UCM “Humanidades, Ciencia y Salud”, Departamento de Enfermería, Facultad de Enfermería, Fisioterapia y Podología, Universidad Complutense de Madrid, 28035 Madrid, Spain; 4 Center Interdisciplinary Research in Health (CIIS), Nursing School (Lisbon), Institute of Health Sciences, Universidade Católica Portuguesa, 1649-023 Lisbon, Portugal; cristina_marques@ucp.pt; 5 Grupo de Investigación en Cuidados (InveCuid), Instituto de Investigación Sanitaria Hospital 12 de Octubre (imas12), 28041 Madrid, Spain; 6 Department of Intensive Care, Hospital Universitario 12 de Octubre, 28041 Madrid, Spain; 7 Centro Municipal de Salud Comunitaria Centro, Madrid Salud, Ayuntamiento de Madrid, 28013 Madrid, Spain; 8 Unidad de Investigación de la Gerencia Asistencial de Atención Primaria, Servicio Madrileño de la Salud, 28035 Madrid, Spain; 9 Academia Central de la Defensa, Escuela Militar de Sanidad, Ministerio de Defensa, 28040 Madrid, Spain

**Keywords:** attitudes, beliefs, health occupations, COVID-19, students, vaccination

## Abstract

It is important to know the attitudes of students of health sciences (SHSs) towards vaccination since they will be tomorrow’s health professionals. Vaccination is a powerful tool in the fight against COVID-19. The aim of the present, cross-sectional study was to examine how the COVID-19 pandemic has influenced the attitude of SHSs towards vaccination. Data were collected in the form of a questionnaire from all students of nursing, physiotherapy and chiropody matriculated at a Madrid University for the academic year 2019/2020 (i.e., before the start of the pandemic [Q1]), and from all those matriculated for the year 2021/22 (i.e., c18 months after the pandemic was declared [Q2]). A multivariate analysis was performed to identify the influence of sex, degree being studied, course year and the time of answering (Q1 or Q2), on the dimensions Beliefs, Behaviours and General Attitude. Overall, 1894 questionnaires were returned (934 [49.3%] for Q1, and 960 [50.7%] for Q2), of which 70.5% were completed by students of nursing, 14% by students of physiotherapy and 15.4% by those studying chiropody. In Q2, the results for all three dimensions were significantly better (*p* < 0.05). The most important influencing factors were being a student of nursing, being in the final years of training (years 3 or 4), female gender and answering at the time of Q2. The results obtained are encouraging since student nurses (who showed Q1 and Q2 General Attitude scores of 3.34 and 3.47 (maximum possible 4), respectively [*p* < 0.05]) are the health professionals of tomorrow most likely to be involved in vaccination programmes.

## 1. Introduction

Despite vaccination being among the most successful means of controlling transmissible diseases [1], recent years have seen the rise of anti-vaccination movements, the rhetoric of which has contributed towards vaccine hesitancy [2,3]—even among health professionals [4,5]. This is particularly worrying, since the latter have a great influence on the patients they see [6,7]. Indeed, it is well known that the beliefs and attitudes of health professionals influence the behaviour of, and decisions taken by, their patients (and their respective families) with regard to vaccination [8,9,10]. Students of the health sciences (SHSs) will be tomorrow’s health professionals, and on their shoulders will lie the responsibility of carrying out future vaccination programmes. Knowing their attitudes towards vaccination is, therefore, important; this might help in the task of allaying their fears and concerns.

A number of international studies have shown that attitudes can vary considerably depending on where, when and how they are examined. For example, reports from Serbia [11], Florida (USA) [12], Australia [13] and central and southern Italy [14] highlight more positive attitudes than others from the USA [15], Germany [16], Canada [17] and Italy (Messina) [18]. In January 2020, our group examined the general attitude of students of nursing, physiotherapy and chiropody (all at the Universidad Complutense de Madrid, Madrid, Spain) towards vaccination [19], and found attitudes to be generally positive, especially among students of nursing, female students and students in their final years. 

The vaccination programme to combat the COVID-19 pandemic, which began to affect Europe in the first quarter of 2020 [20], has been the subject of controversy, with the media, politicians and the general public taking very different positions [21]. Although the papers being published on the safety and effectiveness of the vaccines used have reduced hesitancy in some groups [22], there has also been an “infodemic” with messages designed to misinform and confuse the public [23]. Google Trends data reveal anti-vaccine searches to have risen continuously in number over the course of the pandemic [24], and anti-vaxxers and COVID-deniers have had a notable presence in the media [25]. The aim of the present cross-sectional study was to compare the attitudes of SHSs at a Spanish university towards vaccination before and during the pandemic (18 months in).

## 2. Materials and Methods

The study subjects were 3222 students of nursing, physiotherapy and chiropody at the Universidad Complutense de Madrid (Madrid, Spain), 1582 of whom were matricu-lated for the academic year 2019/2020 (i.e., before the pandemic was declared), and 1640 of whom were matriculated for the year 2021/2022 (i.e., which began c18 months after the pandemic was declared). All matriculated students, irrespective of their course year, were asked to complete the “Attitudes and Behaviour with Regard To Vaccination Among Health Science Students Questionnaire” (ACVECS according to its Spanish initials—Cuestionario sobre Actitudes y Conductas hacia la Vacunación entre Estudiantes de Ciencias de la Salud) [26]) between 15–30 January 2020 (Q1), and 1 October to 22 November 2021 (Q2). Thus, all students completed at least one questionnaire, and some completed both Q1 and Q2. The ACVECS questionnaire is composed of 24 items, the first 15 of which examine the dimension “Beliefs”, and the last nine “Behaviour”. Taken together, these 24 items determine the “General Attitude” towards vaccination [19,26]. In the validation analysis performed by the questionnaire’s authors, ordinal α (a rating of reliability) was 0.92, the Kaiser–Meyer–Olkin index was 0.90, and the Barlet sphericity value was χ2 = 4142.1 (*p* < 0.001). The answer provided for each item is recorded on a five-point Likert scale from 0 = totally disagree, to 4 = totally agree (items 1, 2, 7, 8, 15 and 23 have inverted scoring) [19,26]. Beliefs, Behaviour and General Attitude scores of ≥3 were considered positive (i.e., vaccination favourable), ≤1 negative and 2 neutral or indifferent.

Data were collected during visits to classrooms. The students were told the purpose of the study and that their anonymity was assured. Those students who wished to take part did so over a period of 10–15 min of classroom time using the Google Forms online platform. Sample size requirement analysis showed that 310 respondents were needed for a 5% error and 95% confidence limits, as discussed in previous studies [19]. The data collected were then transferred to a Microsoft Excel 365^®^ database.

Means and standard deviation were calculated for quantitative variables, and frequencies and percentages for qualitative variables. Student’s *t*-test was used to compare quantitative variables, and the chi-squared test to compare qualitative variables. The effect of gender, age, degree being studied, course year (1, 2, 3 or 4) and the time of answering (Q1 or Q2) on Beliefs, Behaviour and General Attitude were examined by multiple linear regression (forward stepwise, with an F probability value of ≤0.05 for entry and >0.10 for exit). Significance was set at *p* ≤ 0.05. All calculations were performed using SPSS^®^ v.25.0 software.

The study was approved by the Research Committee of the Faculty of Nursing, Physiotherapy and Chiropody, Universidad Complutense de Madrid, and by the Ethics in Research Committee of the Hospital Universitario Clínico San Carlos (CI: 20/376-E).

## 3. Results

Of the 3222 students matriculated over the two academic years, 1894 (58.8%) completed the questionnaire, i.e., 934 (49.3%) at Q1, and 960 (50.7%) at Q2. Some 70.5% of the returned questionnaires were provided by students of nursing, 14% by students of physiotherapy and 15.6% by those studying chiropody. Taking into account the number of students studying each of these degrees, the participation by students of nursing was somewhat higher (64.8%, compared with 47.3% for students of physiotherapy, and 48.9% for those studying chiropody). Participation was slightly lower among students studying their final years (Table 1). Women made up 80.9% of all respondents (85.3% of nursing students were female, as were 80.1% of chiropody students and 59.8% of physiotherapy students; *p* < 0.001). The mean age of respondents was 21.4 ± 0.26 years (range 17–60 years). The students of chiropody were a little older (22.0 ± 0.59 years) than those studying nursing (21.5 ± 0.33 years) or physiotherapy (20.6 ± 0.53 years) (*p* = 0.011).

For Q2, the results for the three dimensions measured by the AVECS questionnaire, i.e., Beliefs, Behaviours and General Attitude, were all significantly better (*p* < 0.05) than those recorded for Q1 (Table 2).

Table 3, Table 4 and Table 5 show the change in scores between Q1 and Q2 for all three dimensions measured, with respect to gender, degree being studied and course year. Compared with the Q1 scores, the Q2 scores for students of nursing showed significant improvements in all dimensions (*p* < 0.05). Furthermore, taking students of all three degrees together, Q2 improvements were seen for both men and women, and for students in their first and third years (*p* < 0.05). Q2 improvements were, in fact, seen across the board for students of all degrees, although not always so clearly in each of the dimensions measured. No Q2 scores were poorer than Q1 scores.

Taking the students of all three degrees together, the Q2 scores for 16 of the 24 (66.7%) questionnaire items (nos. 2, 3, 6, 7, 8, 9, 10, 11, 13, 14, 15, 16, 20, 22, 23, 24 in Table 6) were significantly higher than the corresponding Q1 scores. Five (nos. 1, 5, 17, 19, 21) of the remaining eight items also showed higher Q2 scores, but the differences were not significant due to the already high corresponding Q1 scores. No significant difference was seen between the Q1 and Q2 scores for the remaining three items (nos. 4, 12, 18) (Table 6).

Table 7 shows the results of the multivariate analysis, identifying the influencing independent variables and their effect size, for all three AVECS dimensions measured. Being a student of nursing was the variable that most positively influenced the scores for all three, followed by answering at the time of Q2. Being in the fourth (final) year of any degree was also associated with improvements in Beliefs and General Attitude, whereas the female sex was related to better Behaviour and General Attitude. Being in the third year of one’s degree had a positive impact on beliefs.

## 4. Discussion

This is the first work to compare attitudes towards vaccination among SHSs during the present pandemic. The results show that these were generally very positive to begin with, and that they improved during these difficult times. Some students, however, revealed some reticence in their answers to certain questions. For example, 10.1% were doubtful about the effectiveness of vaccination, and 14% questioned its cost-effectiveness. These results, which are similar to those reported by other authors who used the same questionnaire [26], could be conditioned by a poor perception of the risk posed by infectious disease to the young population [27,28], or a lack of knowledge of vaccination in general and vaccination programs in particular [11,12,17]. The fact that the chiropody students do not undertake practical studies at health centres, and considering their—and physiotherapists’—lesser involvement in vaccination programs, might also have influenced the results [19,29].

Previous studies have examined the change in attitude towards vaccination shown by the general population and health professionals. The Vaccine Confidence project reported that, between 2018 and 2020, public confidence in vaccines increased in most European countries, with the populations of Spain and Portugal the most confident of all [30]. In Spain, general confidence in vaccines grew from 64% to 70% (i.e., the percentage of respondents replying positively to [in agreement with] all items in the questionnaire used). The authors of that study point out that these findings could be influenced by people’s perception of the seriousness of the pandemic. Other authors report a significant increase in general confidence in vaccination among the Italian population one year into the pandemic [31]. Similarly, vaccine hesitancy is reported to have diminished in Turkey [32] and Japan [33]. However, in the USA, there has been a general reduction in favourable attitudes towards vaccination over the course of the pandemic [34], with attitudes worsening among Republican voters but remaining stable among Democrat voters. Other studies, performed between March and August 2020, have shown growing reticence towards being vaccinated against COVID-19 among British and Irish populations [35].

With respect to healthcare personnel, Ledda et al. report attitudes to have improved among professionals at an Italian hospital over the course of the pandemic [36]. Della Polla et al. also report Italian workers (including healthcare workers) and students at the University of Naples to have shown a clear improvement in their attitude towards vaccination, and positive change towards employing general preventive measures against COVID-19, including the use of masks, washing hands and social distancing [37].

Several other studies report that the pandemic has changed the attitude of the general population [38], and of healthcare professionals, towards vaccination against influenza [39,40], perhaps due to an increased consciousness of airborne transmission [41].

In the present work, the factors that most influenced having a positive attitude were being a student of nursing, answering at the time of Q2, female gender and being in recent years of study.

The influence of being a student of nursing rather than another discipline (stronger even than the time of answering the questionnaire [Q1 or Q2]) may be explained by the fact that nurses are more involved and have greater responsibilities in vaccination programmes [29] and receive more information about, and training in, vaccination than the students of the other present degrees, and the correlation that exists between knowledge and attitude towards vaccination [11,15,42]. Other studies have reported the good disposition of students of nursing towards vaccination [26,42]. In fact, in a study performed in seven European countries, Spanish students of nursing showed the strongest disposition towards vaccination against COVID-19, followed by those studying in Italy [43].

After adjusting for age, sex and degree type, etc., answering at the time of Q2 was related to improvements in the measured dimensions, supporting the hypothesis that the pandemic has had a positive effect on attitude towards vaccination.

The students in their final years showed a better attitude towards vaccination, a phenomenon identified in earlier studies from Spain [26,42] and other countries [11,44,45,46]. This is probably related to the greater knowledge acquired and capacities developed by students further on in their studies. It should be noted that recent studies on vaccination against COVID-19 report the same [47,48]. In fact, non-health science students with greater knowledge of the COVID-19 vaccines are also reported to show better attitudes [14]. The strength of the relationship between deeper knowledge and acceptance of vaccination may be influenced by the effectiveness of information campaigns [14,30].

The better results seen for female students in the present work agree with previously reported studies [49,50,51]. It may be that women show greater solidarity [52], or that since male students of nursing are reduced in number, they may more often decline to take part in group activities that might influence their attitudes [53].

The good—and later, further improved—attitude towards vaccination seen in the present work is important since many studies concur that such attitudes improve the likelihood of requesting the influenza [54,55,56,57] and COVID-19 vaccines [43,48,58].

Finally, the present work has a number of limitations. The sample, although large, may not be entirely representative of SHSs at the national level; further studies at other universities are required to confirm the results. Moreover, the data used in analyses came from self-answered questionnaires, and such data can be affected by respondents’ willingness to please.

## 5. Conclusions

The overall attitude of the present students towards vaccination was good, and improved significantly during the first 18 months of the pandemic, perhaps in part because of the education received and in part as a result of living through these times with their attendant fears and worries. The results show that attitudes can change for the better, reinforcing the need to promote health and increase the public’s knowledge regarding vaccines and vaccination.

The fact that the COVID-19 vaccines can protect people from the worst of the disease, plus the information campaign surrounding vaccination against COVID-19, has probably improved people’s perception of vaccination in general, making them more likely to accept vaccination against other diseases.

Further studies in other populations of health sciences students are required, both nationally and internationally. It will also be important to determine whether the improvements seen in this work are maintained after the pandemic has finally ended.

## Figures and Tables

**Table 1:** Participation in terms of degree being pursued and course year (Q1, Q2 and Total).

Survey	Degree	Total Matriculated	Total Participating Subjects	Participating Subjects by Course Year			
				1°	2°	3°	4°
		n (%)	n (%)	n (%)	n (%)	n (%)	n (%)
Q1 (Jan/2020)	Nursing	1039 (65.6)	624 (66.9)	220 (35.2)	192 (30.8)	120 (19.3)	92 (14.7)
	Physiotherapy	262 (16.6)	162 (17.3)	40 (24.6)	50 (30.8)	30(18.5)	42 (25.9)
	Chiropody	281 (17.8)	148 (15.8)	32 (21.6)	58 (39.2)	28 (18.9)	30 (20.3)
	Total	1582 (100.0)	934 (100.0)	292 (31.2)	300 (32.1)	178 (19.2)	164 (17.5)
Q2 (OCT/NOV 2021)	Nursing	1024 (62.4)	712 (74.2)	171 (24.0)	150 (21.1)	194 (27.2)	197 (27.7)
	Physiotherapy	300 (18.3)	104 (10.8)	42 (40.4)	29 (27.9)	18(17.3)	15 (14.4)
	Chiropody	316 (19.3)	144 (15.0)	42 (29.2)	30 (20.8)	41 (28.5)	31 (21.5)
	Total	1640 (100.0)	960(100.0)	255 (26.6)	209 (21.8)	253 (26.4)	243 (25.3)
TOTAL (Q1/Q2)	Nursing	2063 (64.0)	1336 (70.5)	391 (29.3)	342 (25.6)	314 (23.5)	289 (21.6)
	Physiotherapy	562 (17.4)	266 (14.0)	82 (30.8)	79 (29.7)	48(18.0)	57 (21.4)
	Chiropody	597 (18.5)	292 (15.4)	74 (25.0)	88 (29.7)	69 (23.3)	61 (20.6)
	Total	3222 (100.0)	1894 (100.0)	547 (28.9)	509 (26.9)	431 (22.8)	407 (21.5)

**Table 2:** Distribution of scores for the ACVECS questionnaire dimensions (Q1 and Q2).

Survey	Beliefs Mean ± SD	*p* Value	Behaviour Mean ± SD	*p* Value	General Attitude Mean ± SD	*p* Value
Q1(ENE/2020)	3.23 ± 0.47	0.001	3.35 ± 0.51	0.009	3.27 ± 0.46	0.006
Q2 (OCT_NOV/2021)	3.38 ± 0.45		3.47 ± 0.47		3.41 ± 0.43	

**Table 3:** Distribution of Q1 and Q2 scores for the Beliefs dimension of the ACVECS questionnaire with respect to gender, degree being studied, and course year for each degree and as a whole for all degrees taken together.

Variables	Categories	Beliefs Q1 Mean ± SD	Beliefs Q2 Mean ± SD	*p* Value
Sex	Male	3.15 ± 0.46	3.34 ± 0.51	0.001
	Female	3.24 ± 0.50	3.39 ± 0.43	*p* < 0.0001
Degree	Nursing	3.28 ± 0.43	3.44 ± 0.41	*p* < 0.0001
	Physiotherapy	3.07 ± 0.51	3.22 ± 0.51	0.023
	Chiropody	3.14 ± 0.53	3.23 ± 0.50	0.161
Course year for all degrees taken together	1st	3.17 ± 0.48	3.34 ± 0.41	*p* < 0.0001
	2nd	3.22 ± 0.46	3.33 ± 0.47	0.009
	3rd	3.23 ± 0.49	3.44 ± 0.45	*p* < 0.0001
	4th	3.33 ± 0.46	3.42 ± 0.45	0.063
Nursing course year	1st	3.19 ± 0.45	3.34 ± 0.38	0.001
	2nd	3.29 ± 0.42	3.38 ± 0.42	0.050
	3rd	3.32 ± 0.39	3.51 ± 0.40	*p* < 0.0001
	4th	3.42 ± 0.42	3.47 ± 0.41	0.407
Physiotherapy course year	1st	3.07 ± 0.42	3.26 ± 0.49	0.068
	2nd	3.02 ± 0.48	3.01 ± 0.61	0.952
	3rd	2.88 ± 0.64	3.46 ± 0.35	0.001
	4th	3.26 ± 0.45	3.20 ± 0.42	0.695
Chiropody course year	1st	3.13 ± 0.66	3.38 ± 0.39	0.051
	2nd	3.13 ± 0.48	3.34 ± 0.47	0.060
	3rd	3.20 ± 0.53	3.05 ± 0.51	0.241
	4th	3.12 ± 0.50	3.16 ± 0.58	0.815

**Table 4:** Distribution of Q1 and Q2 scores for the Behaviour dimension of the ACVECS questionnaire with respect to gender, degree being studied, and course year for each degree and as a whole for all degrees taken together.

Variables	Categories	Behaviour Q Mean ± SD	Behaviour Q2 Mean ± SD	*p* Value
Sex	Male	3.25 ± 0.53	3.36 ± 0.54	0.040
	Female	3.38 ± 0.50	3.49 ± 0.45	*p* < 0.0001
Degree	Nursing	3.42 ± 0.45	3.52 ± 0.44	*p* < 0.0001
	Physiotherapy	3.16 ± 0.62	3.30 ± 0.54	0.061
	Chiropody	3.29 ± 0.57	3.36 ± 0.53	0.264
Course year for all degrees taken together	1st	3.38 ± 0.50	3.46 ± 0.44	0.049
	2nd	3.34 ± 0.51	3.41 ± 0.52	0.134
	3rd	3.29 ± 0.58	3.49 ± 0.47	*p* < 0.0001
	4th	3.39 ± 0.47	3.50 ± 0.47	0.026
Nursing course year	1st	3.40 ± 0.46	3.48 ± 0.43	0.125
	2nd	3.43 ± 0.43	3.48 ± 0.48	0.370
	3rd	3.35 ± 0.48	3.55 ± 0.43	*p* < 0.0001
	4th	3.50 ± 0.41	3.55 ± 0.42	0.407
Physiotherapy course year	1st	3.25 ± 0.59	3.43 ± 0.50	0.164
	2nd	3.10 ± 0.60	3.05 ± 0.61	0.698
	3rd	3.02 ± 0.79	3.49 ± 0.43	0.029
	4th	3.23 ± 0.48	3.20 ± 0.46	0.836
Chiropody course year	1st	3.38 ± 0.60	3.44 ± 0.38	0.601
	2nd	3.25 ± 0.55	3.44 ± 0.50	0.122
	3rd	3.26 ± 0.63	3.23 ± 0.54	0.826
	4th	3.29 ± 0.50	3.35 ± 0.70	0.692

**Table 5:** Distribution of Q1 and Q2 scores for the General Attitude dimension of the ACVECS questionnaire with respect to gender, current degree, and course year for each degree and as a whole for all degrees taken together.

Variables	Categories	General Attitude Q1 Mean ± SD	General Attitude Q2 Mean ± SD	*p* Value
Sex	Male	3.19 ± 0.49	3.35 ± 0.50	0.003
	Female	3.30 ± 0.45	3.43 ± 0.41	*p* < 0.0001
Degree	Nursing	3.34 ± 0.41	3.47 ± 0.39	*p* < 0.0001
	Physiotherapy	3.11 ± 0.52	3.25 ± 0.50	0.026
	Chiropody	3.20 ± 0.52	3.28 ± 0.49	0.176
Course year for all degrees taken together	1st	3.25 ± 0.46	3.38 ± 0.39	*p* < 0.0001
	2nd	3.26 ± 0.44	3.36 ± 0.47	0.021
	3rd	3.25 ± 0.49	3.46 ± 0.43	*p* < 0.0001
	4th	3.35 ± 0.43	3.45 ± 0.44	0.035
Nursing course year	1st	3.27 ± 0.43	3.39 ± 0.37	0.005
	2nd	3.34 ± 0.38	3.42 ± 0.42	0.096
	3rd	3.33 ± 0.39	3.53 ± 0.38	*p* < 0.0001
	4th	3.45 ± 0.39	3.50 ± 0.39	0.379
Physiotherapy course year	1st	3.14 ± 0.46	3.32 ± 0.48	0.085
	2nd	3.05 ± 0.49	3.03 ± 0.58	0.837
	3rd	2.94 ± 0.66	3.47 ± 0.33	0.003
	4th	3.25 ± 0.43	3.21 ± 0.42	0.735
Chiropody course year	1st	3.22 ± 0.62	3.40 ± 0.36	0.130
	2nd	3.17 ± 0.48	3.38 ± 0.45	0.067
	3rd	3.22 ± 0.55	3.12 ± 0.51	0.413
	4th	3.18 ± 0.45	3.23 ± 0.60	0.752

**Table 6:** Frequencies and percentages of students showing positive and negative scores on the 24 items of the ACVECS questionnaire.

Questionnaire Items	SURVEY	Disagree n (%)	ND/NA n (%)	Agreed n (%)	*p* Value
1. I have doubts about the effectiveness of vaccines	Q1	750 (80.3)	80 (8.6)	104 (11.1)	0.261
	Q2	797 (83.0)	76 (7.9)	87 (9.1)	
2. I would rather have influenza than be vaccinated against it	Q1	703 (75.3)	135 (14.5)	96 (10.3)	*p* < 0.001
	Q2	814 (84.8)	90 (9.4)	56 (5.8)	
3. I am convinced that marketed vaccines are safe	Q1	73 (7.8)	151 (16.2)	710 (76.0)	*p* < 0.001
	Q2	49 (5.1)	100 (10.4)	811 (84.5)	
4. I am interested in learning more about vaccination	Q1	71 (7.6)	198 (21.2)	665 (71.2)	0.693
	Q2	66 (6.9)	216 (22.5)	678 (70.6)	
5. I believe it important to check my vaccination status before travelling to a tropical country such as Mexico or Thailand	Q1	7 (0.7)	23 (2.5)	904 (96.8)	0.192
	Q2	9 (0.9)	13 (1.4)	938 (97.7)	
6. National and international vaccine campaigns are cost-effective	Q1	146 (15.6)	300 (32.1)	488 (52.2)	*p* < 0.001
	Q2	120 (12.5)	221 (23.0)	619 (64.5)	
7. It is not worth being vaccinated against a disease for which effective treatment exists	Q1	786 (84.2)	92 (9.9)	56 (6.0)	*p* < 0.001
	Q2	868 (90.4)	66 (6.9)	26 (2.7)	
8. Vaccinating the adult population is not important	Q1	882 (94.4)	22 (2.4)	30 (3.2)	0.001
	Q2	939 (97.8)	7 (0.7)	14 (1.5)	
9. Health science students are ethically obliged to be vaccinated against influenza	Q1	146 (15.6)	217(23.2)	571 (61.1)	0.002
	Q2	107 (11.1)	199 (20.7)	654 (68.1)	
10. Being vaccinated myself has a positive influence on the behaviour of my patients	Q1	51 (5.5)	169 (18.1)	714 (76.4)	*p* < 0.001
	Q2	29 (3.0)	81 (8.4)	850 (88.5)	
11. Students should be vaccinated to reduce the transmission of infectious diseases in hospitals	Q1	22 (2.4)	55 (5.9)	857 (91.8)	*p* < 0.001
	Q2	11 (1.1)	26 (2.7)	923 (96.1)	
12. I should review my vaccination status before starting clinical training	Q1	28 (3.0)	112 (12.0)	794 (85.0)	0.132
	Q2	36 (3.8)	141 (14.7)	783 (81.6)	
13. I should be vaccinated against influenza every year, even it means missing hours of practical training	Q1	159 (17.0)	228 (24.4)	547 (58.6)	*p* < 0.001
	Q2	95 (9.9)	187 (19.5)	678 (70.6)	
14. I would be vaccinated irrespective of what my peers might do	Q1	25 (2.7)	50 (5.4)	859 (92.0)	*p* < 0.001
	Q2	15 (1.6)	20 (2.1)	925 (96.4)	
15. If I am in good health there is no need to be vaccinated	Q1	750 (80.3)	129 (13.8)	55 (5.9)	*p* < 0.001
	Q2	866 (90.2)	52 (5.4)	42 (4.4)	
16. I would recommend my patients adhere to the established vaccination calendar	Q1	8 (0.9)	36 (3.9)	890 (95.3)	0.002
	Q2	5 (0.5)	13 (1.4)	942 (98.1)	
17. I would inform my patients of the effectiveness, indications and side effects of each vaccine	Q1	6 (0.6)	29 (3.1)	899 (96.3)	0.431
	Q2	5 (0.5)	21 (2.2)	934 (97.3)	
18. I would travel to a tropical country only after consulting Spain’s International Vaccination about the vaccines I require	Q1	35 (3.7)	51 (5.5)	848 (90.8)	0.428
	Q2	40 (4.2)	65 (6.8)	855 (89.1)	
19. I would be vaccinated against HIV when a vaccine becomes available and when shown to be acceptably safe and effective	Q1	32 (3.4)	74 (7.9)	828 (88.7)	0.762
	Q2	28 (2.9)	72 (7.5)	860 (89.6)	
20. If being vaccinated against influenza were readily accessible to me, I would be vaccinated every year	Q1	64 (6.9)	120 (12.8)	750 (80.3)	0.007
	Q2	49 (5.1)	88 (9.2)	823 (85.7)	
21. I would be vaccinated against anything my doctor recommends, even if I have to pay for it	Q1	87 (9.3)	261 (27.9)	586 (62.7)	0.545
	Q2	101 (10.5)	252 (26.3)	607 (63.2)	
22. When I begin work at a hospital, I will make sure I am vaccinated against everything preventable	Q1	10 (1.1)	56 (6.0)	868 (92.9)	0.022
	Q2	10 (1.0)	32 (3.3)	918 (95.6)	
23. I would only be vaccinated in exceptional circumstances (epidemics, health alerts, etc.	Q1	683 (73.1)	139 (14.9)	112 (12.0)	*p* < 0.001
	Q2	826 (86.0)	89 (9.3)	45 (4.7)	
24. I will be vaccinated against influenza every year I have clinical training	Q1	122 (13.1)	229 (24.5)	583 (62.4)	*p* < 0.001
	Q2	61 (6.4)	175 (18.2)	724 (75.4)	

**Table 7:** Multivariate analysis: relationships shown by the dimensions Beliefs, Behaviour and General Attitude with the variables sex, age, degree being pursued, and course year.

Dependent Variable	Independent Variables	Unstandardised Coefficients B (95%CI)	Standardised Coefficients	*p* Value
Beliefs	Constant	2.938 (2.868–3.008)		*p* < 0.001
	Nursing	0.193 (0.148–0.237)	0.189	*p* < 0.001
	Survey: Q2	0.128 (0.087–0.169)	0.137	*p* < 0.001
	Course Year 4th	0.103 (0.051–0.155)	0.091	*p* < 0.001
	Course Year 3rd	0.069 (0.019–0.120)	0.063	0.007
Behaviour	Constant	3.059 (2.975–3.144)		*p* < 0.001
	Nursing	0.173 (0.124–0.222)	0.159	*p* < 0.001
	Q2	0.101 (0.057–0.145)	0.101	*p* < 0.001
	Female gender	0.098 (0.042–0.155)	0.078	0.001
General attitude	Constant	2.974 (2.898–3.049)		*p* < 0.001
	Nursing	0.183 (0.139–0.227)	0.186	*p* < 0.001
	Q2	0.120 (0.081–0.160)	0.134	*p* < 0.001
	Course Year 4th	0.067 (0.019–0.115)	0.061	*p* < 0.001
	Female gender	0.058 (0.007–0.108)	0.050	0.026

## Data Availability

Not applicable.
